# Wide-Bandgap Semiconductors for Radiation Detection: A Review

**DOI:** 10.3390/ma17051147

**Published:** 2024-03-01

**Authors:** Ivana Capan

**Affiliations:** Ruđer Bošković Institute, Bijenička 54, 10000 Zagreb, Croatia; capan@irb.hr

**Keywords:** wide-bandgap semiconductors, radiation, detectors

## Abstract

In this paper, an overview of wide-bandgap (WBG) semiconductors for radiation detection applications is given. The recent advancements in the fabrication of high-quality wafers have enabled remarkable WBG semiconductor device applications. The most common 4H-SiC, GaN, and β-Ga_2_O_3_ devices used for radiation detection are described. The 4H-SiC and GaN devices have already achieved exceptional results in the detection of alpha particles and neutrons, thermal neutrons in particular. While β-Ga_2_O_3_ devices have not yet reached the same level of technological maturity (compared to 4H-SiC and GaN), their current achievements for X-ray detection indicate great potential and promising prospects for future applications.

## 1. Introduction

The need for reliable and efficient radiation detectors for particle physics, space technologies, nuclear power plants, medicine, and homeland security applications is growing rapidly. The requirements set for radiation detectors are complex, from exceptional efficiency and energy resolution to extreme radiation tolerance. Among numerous candidates, semiconductor radiation detectors offer plenty of advantages due to their exceptional material properties. For many decades, Si-based radiation detectors have been the champions in the radiation detection arena [1]. However, Si-based devices are reaching the limit of their performance, and it is dubious that significant improvements will follow in the years to come. Due to the wider bandgap (compared to Si 1.12 eV, for example) and the recent astonishing progress in material fabrication, wide-bandgap (WBG) semiconductors are becoming a new driving force for radiation detection. Material properties of WBG semiconductors (Table 1) that are factors in radiation detection applications and make WBG semiconductors suitable for high-temperature and radiation-harsh environments are, among others, a wide bandgap, high breakdown voltage, high electron mobility, and exceptional thermal properties [2].

The list of the most scrutinized WBG semiconductors includes, but is not limited to, silicon carbide (SiC), gallium nitride (GaN), gallium arsenide (GaAs), cadmium telluride (CdTe), and gallium oxide (Ga_2_O_3_). In this review paper, attention will primarily be focused on the selected materials, namely, SiC, GaN, and Ga_2_O_3_—more precisely, 4H-SiC and β-Ga_2_O_3_. The reasoning is the following, the 4H polytype is the preferred material among the best-known SiC polytypes (2C-, 3C-, 4H-, and 6H-SiC) for electronic components due to the high and isotropic mobility of charge carriers [2,3]. Monoclinic β-Ga_2_O_3_ is the most stable among the five crystalline phases of Ga_2_O_3_ single crystals (α, β, γ, δ, and ε phases) and poses a very high breakdown electric field (Table 1) [4]. The basic material properties for 4H-SiC, GaN, and β-Ga_2_O_3_ are given in Table 1.

**Table 1:** Material properties for the selected wide-bandgap (WBG) semiconductors [4,5].

	GaN	4H-SiC	β-Ga_2_O_3_	GaAs	CdTe
Crystal structure	Wurtzite	Wurtzite	Monoclinic	Zinc blende	Zinc blende
Relative dielectric constant ε	9.0	9.7	10.2–12.4	13.1	10.3
Bandgap energy (eV)	3.4	3.3	4.5	1.424	1.44
Density (g/cm^3^)	6.15	3.21	6.44	5.32	5.85
e-h pair creation energy (eV)	8.9	7.8	15.6 [6]	4.2	4.43
Breakdown electric field (MV/cm)	3.3	2.5	7	0.4	--
Electron mobility (cm^2^/Vs)	1200	1000	~200	8500	1100
Thermal conductivity (W/cmK)	2.1	2.7	0.11–0.27	0.55	0.06

**Figure 1:**
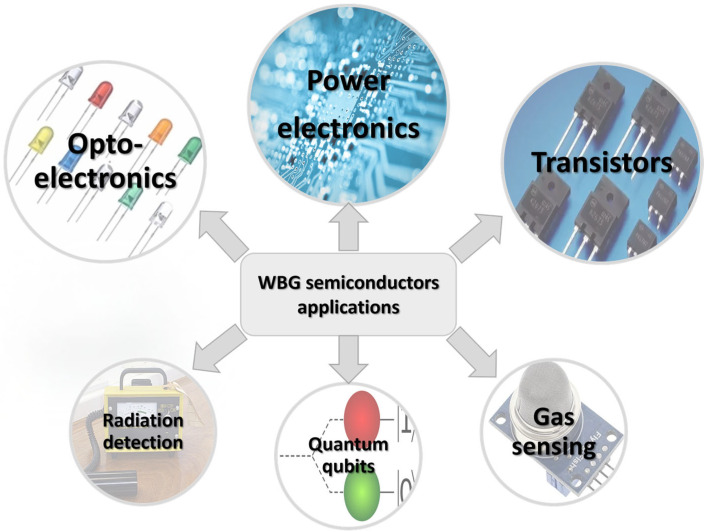
Diagram representing areas for the WBG semiconductor devices applications.

Figure 1 shows several areas where WBG semiconductors are used. Due to the material properties (Table 1), WBG semiconductors are dominantly used for applications in power electronics [7,8,9]. However, the number of new applications is continuously increasing, and WBG semiconductors are becoming the material of special interest for quantum technology [10,11,12], gas sensing [13], and radiation detection [14,15,16,17,18,19].

The main aim of this review paper is to provide an easy-to-follow yet practical and, above all, useful overview of the recent achievements of WBG semiconductors used for radiation detection. The paper is explicitly focused on radiation detection applications. Other important aspects such as crystal growth, material characterization, or different applications will not be covered. It should be noted that each aspect such as crystal growth or comprehensive material characterization (structural, morphological, electrical, etc.) deserves a dedicated review paper. Therefore, the appropriate references that could be useful for additional reading and better understanding of the subject will be provided whenever relevant. We sincerely recommend that our readers look for more specific details in the provided references and references therein.

The structure of this paper is the following. Three main sections (Section 2, Section 3 and Section 4) are devoted to the selected WBG semiconductors. Namely, Section 2 to 4H-SiC, Section 3 to GaN, and Section 4 to β-Ga_2_O_3_. Each section has two main subsections. The first one will provide a brief introduction, and the second one will provide an overview of the recent advancements, covering radiation response to different radiation sources such as alpha particles, neutrons, and X-rays. Section 5 provides a summary of the main challenges and future perspectives.

## 2. 4H-SiC

Research on SiC dates back to the end of the 19th century when SiC was recognized as a material for an abrasive powder and refractory bricks [7]. In the 1950s, the SiC potential was again recognized, this time for high-temperature electronic devices [7]. Despite certain efforts through the decades that followed, research on SiC began to flourish by the end of the 20th century. The progress in the fabrication of high-quality 4H-SiC wafers has enabled remarkable 4H-SiC-based device applications: power electronics [8,9], quantum sensing [10,11,12], and radiation detection [14,15,16,17,18,19]. This has influenced the significant increase in the market value. The SiC device market, valued at around USD 2 billion in 2023, is projected to increase from USD 11 billion to USD 14 billion in 2030 [20].

**Figure 2:**
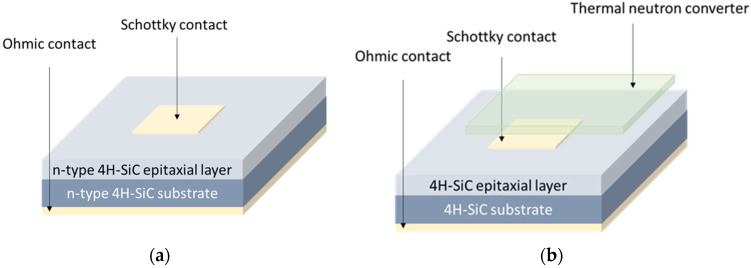
(**a**) A scheme of the typical n-type 4H-SiC SBD used for radiation detection and (**b**) a scheme of 4H-SiC SBD with the additional thermal neutron converter placed above the Schottky contact. The converter is used for thermal neutron detection and is usually placed a few mm above the Schottky contact.

There is a whole variety of 4H-SiC-based devices that are currently being used as radiation detectors. The most common are PiN diodes [21], metal-oxide-semiconductor (MOS) structures [22,23], and Schottky barrier diodes (SBDs) [16,17,18]. Even though the SBD is one of the simplest devices, it has many advantages, and it has been chosen as the preferred structure in many studies [24]. Figure 2a shows a scheme of a typical n-type 4H-SiC SBD. In lots of reported studies, Ni is a preferred material for Schottky and Ohmic contacts for the n-type SBDs. However, it should be noted that other metals are also being used. Osvald et al. [25] have recently reported on Ni/Au Schottky contacts and Chen et al. [26] on the possible benefits of Mo Schottky contacts. Lees et al. [27] have made additional changes and used semi-transparent Cr/Ni Schottky contacts. As we shall explain later in the text, semi-transparent Schottky contacts could improve the efficiency for the detection of low-energy X-rays, compared to conventional Schottky contacts. More detailed information about the key parameters of 4H-SiC SBDs, such as the epitaxial layer thickness and Schottky contact area, could be found elsewhere [24]. In addition, a list of excellent review papers dedicated to SiC has recently been published. De Napoli [28] and Coutinho et al. [3] have provided extensive overviews of crystal growth, material properties, and characterization techniques, with a dedicated focus on SiC radiation detection applications.

### 2.1. Radiation Response to Alpha Particles and Neutrons

Almost all research on the radiation response starts with laboratory tests using alpha particles from ^241^Am source. 4H-SiC-based devices are not an exception. The early and yet still significant work in the area of 4H-SiC radiation response to alpha particles was conducted by Ruddy et al. [15]. SBDs were fabricated on nitrogen-doped (1 × 10^14^ cm^−3^) 4H-SiC epitaxial layers, 100 µm thick. Schottky contacts Au/Pt/Ti were deposited by electron beam evaporation. Using the various alpha emitters such as ^148^Gd, ^238^Pu, ^225^Ac, ^221^Fr, ^217^At, and ^213^Po in the 3.18–8.38 MeV energy range, an excellent energy resolution was achieved. Through the years, progress in energy resolution and efficiency has been reported by many authors [29,30,31,32]. Chaudhuri et al. [32] achieved an excellent energy resolution of 0.29% at the full width at half maximum (FWHM) for 5.48 MeV alpha particles using a 20 µm thick 4H-SiC epitaxial layer. The Schottky contact was Ni/Au, while the Schottky contacts were 2.9 mm or 3.9 mm in diameter. Additionally, the same group has applied 4H-SiC-based MOS structures and compared them to 4H-SiC SBDs. They have reported the highest energy resolution ever measured on SiC-based MOS detectors: 0.42% for 5.48 MeV alpha particles [22].

**Figure 3:**
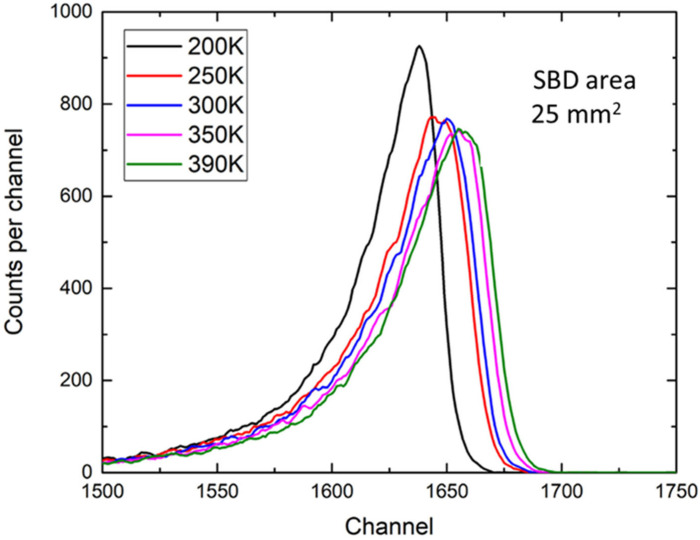
Radiation response of 4H-SiC SBDs (active area of 25 mm^2^) to alpha particles (^241^Am source) in a vacuum at different temperatures. Data reproduced from Ref. [33].

While most of the radiation tests are performed at room temperature (RT), Bernat et al. [33] have recently investigated the 4H-SiC SBDs radiation response to alpha particles at elevated temperatures in a vacuum. They have used n-type 4H-SiC SBDs with a 25 µm thick 4H-SiC epitaxial layer and an active area of 25 mm^2^. Figure 3 shows the response to alpha particles (^241^Am source with a characteristic alpha particle maximum of 5.48 MeV) measured at different temperatures (200–390 K) in a cryostat under a vacuum (<0.1 mbar) [34]. As seen in Figure 3, the peak maximum is shifted on the x-scale as the radiation temperature increases. The estimated energy resolution was 2.5% and did not change with the temperature.

We have summarized some of the most relevant radiation response results of 4H-SiC SBDs to alpha particles in Table 2. The SBD parameters such as the Schottky contact and 4H-SiC epitaxial layer thickness are provided.

Due to their specific material properties, WBG semiconductors are suitable for radiation harsh environments, such as the International Thermonuclear Experimental Reactor (ITER) [33]. The significant advantage of SiC lies in the fact that SiC can detect and distinguish both thermal and fast neutrons. The detection of thermal and fast neutrons by SiC-based devices differs, as thermal neutrons could not be directly detected. Thermal neutron presence is obtained from the detection of ionizing neutron reaction products, such as alpha particles and tritons. In contrast, fast neutrons could be directly detected due to the elastic scattering of fast neutrons with Si or C atoms, or indirectly using polyethylene-based converters. Possible neutron-induced reactions with Si and C that could participate in the 4H-SiC detector response are ^12^C(n,n)^12^C and ^28^ Si(n,n)^28^Si [35]. The probability of this scattering increases as the detector’s active layer thickness increases. Currently, the highest layer thickness is 250 µm as reported by Kleppinger et al. [17]. SBDs with Ni as a Schottky contact were fabricated on 250 µm thick epitaxial layers. An energy resolution of 0.5% FWHM using a ^241^Am source was achieved. Unfortunately, radiation response to fast neutrons has not been measured. Very thick (>300 µm) high-quality 4H-SiC epitaxial layers used for fast neutron detection have not yet been reported. It is reasonable to expect that further advances will be made with an increase in the thicknesses of high-quality epitaxial layers.

The prospect of detecting 14 MeV fast neutrons by 4H-SiC detectors was demonstrated by F.H. Ruddy et al. [36]. Fast neutron response measurements were reported for radiation detectors based on large-volume 4H-SiC SiC pin diodes. Several reaction peaks associated with 14 MeV neutron reactions with the silicon and carbon nuclides in the pin diode were observed. Another work also worth mentioning is that by Flamming et al. [19], who measured the radiation response to fast neutrons using the 100 µm thick 4H-SiC SBD with and without polyethylene converters. Fission neutrons were simulated by using a 2.5 MeV deuterium-deuterium (D-D) neutron generator. As anticipated, better results are achieved using the polyethylene converters.

Hitherto, 4H-SiC SBDs have mostly been used for thermal neutron detection. As already said, they cannot be directly measured; therefore, effective thermal neutron converters are needed. The requirement for such converters is that they are rich in isotopes with a large cross-section for neutrons with energy in the range of k_B_T at RT (k_B_ is the Boltzmann constant). The frequently used converters are ^6^Li and ^10^B [37]. Figure 2b shows a typical set-up for thermal neutron detection using the 4H-SiC SBD with the thermal neutron converter placed just above the Schottky contact (a few mm above). The converter has been horizontally shifted in Figure 2b, for clarity.

**Figure 4:**
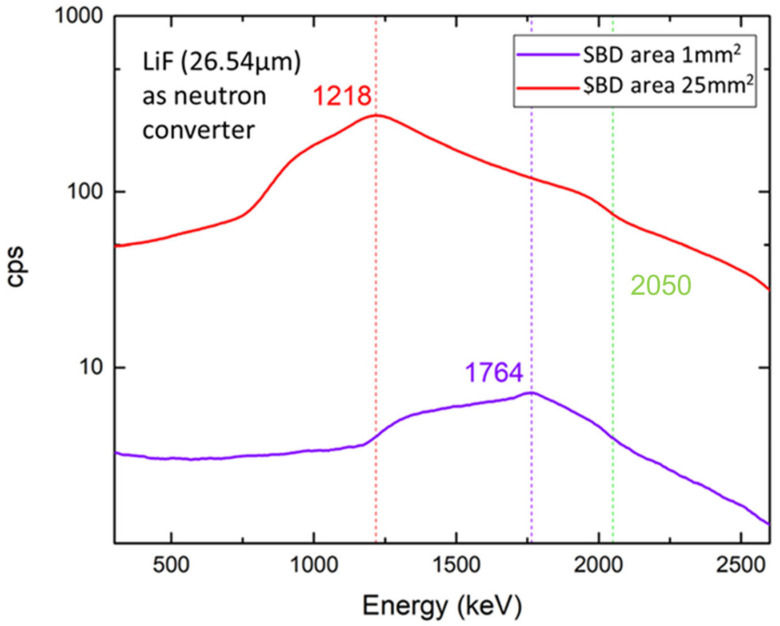
Radiation response of two 4H-SiC SBDs with different active areas (1 mm^2^ and 25 mm^2^). The 26.54 µm thick ^6^LiF thermal neutron converter layer was placed above the SBDs, as already described in the text. Data were taken from Ref. [33].

The best-reported efficiencies for thermal neutron detection using the 4H-SiC devices are between 4 and 5% [38,39,40]. Recently, Bernat et al. [33] have reported on the effects of large-area 4H-SiC SBDs on the radiation response to thermal neutrons. Two different diode areas were compared: 1 mm^2^ and 25 mm^2^. SBDs were fabricated using a 25 µm thick 4H-SiC epitaxial layer and Ni as a Schottky contact. An efficiency of 5.02% with the use of a 26.54 μm thick ^6^LiF thermal neutron converter layer is reported (Figure 4). Additionally, they have shown that with the increase in the SBD active area, the detector could register thermal neutrons with a nuclear reactor power as low as 1 kW.

Contrary to the alpha particles and neutrons, the low-energy X-ray and γ-ray detection by 4H-SiC devices has not yet reached the same level of efficiency. However, several attempts have been made, and they should be noted. Puglisi et al. [41] studied 4H-SiC microstrip detectors for soft X-ray (<20 keV) detection. They achieved an energy resolution of about 700 and 1300 eV FWHM for 1 mm^2^ and 10 mm^2^ detectors measured at RT, respectively. Mandal et al. [42] have achieved an FWHM of 1.2 keV at 59.6 keV using the 50 µm thick 4H-SiC SBDs. Moreover, using the same SBDs, they were able to detect low-energy X-rays in the energy range of 13.93–26.20 eV. Lees et al. [27] have used a slightly different SBD structure. They have reduced the thickness of the Schottky contact (Ni/Ti was used), from a typical 50–100 nm down to 18 nm, and prepared so-called semi-transparent SBD. Different X-ray sources were used: ^55^Fe and ^109^Cd. With 4H-SiC SBD, an energy resolution of 1.47 keV FWHM at 22 keV at RT was reported. Lioliou et al. [43] fabricated photon-counting detectors for X-ray and gamma-ray spectroscopy using the 35 µm thick 4H-SiC SBDs with Mo as a Schottky contact. An energy resolution of 1.67 keV FWHM at 5.9 keV and 1.6 keV FWHM at 59.54 keV was achieved.

As demonstrated in different studies on X-ray detection, energy resolution can be increased by additional modifications on the Schottky contacts (thickness, area, and metal) for 4H-SiC SBDs.

## 3. GaN

**Figure 5:**
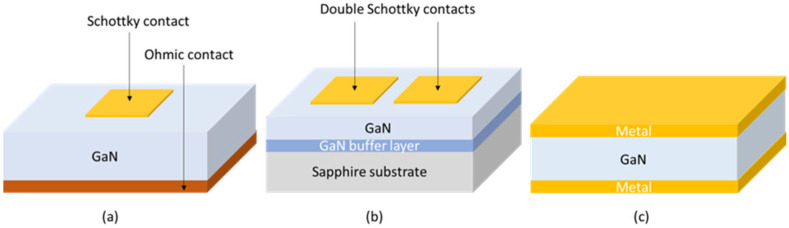
Schemes of the typical GaN devices used for the detection of alpha particles and neutrons, (**a**) SBD, (**b**) double SBD, and (**c**) MSM. Figure adapted from Ref. [5].

Like its WBG counterpart SiC, GaN is a well-known material as the research on GaN dates to the 19th century. Gallium and its compounds were discovered by Paul-Émile Lecoq de Boisbaudran in 1875 [44]. The GaN research flourished in 1969 when H.P. Maruska and J.J. Tietjen reported on the growth of single-crystal film of GaN [45], and again in 1972, when J.I. Pankove, E.A. Miller, and J.E. Berkeyheiser developed a GaN-based blue light detector [46]. The number of GaN-based devices for optoelectronics, power electronics, and radio frequency (RF) applications is constantly growing [45], which leads to a GaN device market value increase. Radiation detection applications are still far less exploited, compared to power electronics. Figure 5 shows a few GaN devices used for radiation detection, such as (a) SBD, (b) double SBD, and (c) metal-semiconductor-metal (MSM). As it is for n-type 4H-SiC, the most common Schottky contacts are Au and Ni. The list of other GaN devices used for radiation detection but not shown here includes PN and PiN diodes [5]. More detailed information about GaN material properties and radiation detection could be found in a comprehensive review paper by Wang et al. [5] and references therein. Additional information about the GaN crystal growth is given in review papers by Denis et al. [47] and Musemeci et al. [48].

### Radiation Response to Alpha Particles and Neutrons

The pioneering work on alpha particle detection was conducted by Vaikuts et al. [49]. A 2 µm thick GaN epitaxial layer was grown on a sapphire substrate. Under the epitaxial layer is a highly doped GaN buffer layer. Two Au Schottky contacts were deposited on the top of the GaN epitaxial layer (Figure 5b). They have achieved charge collection efficiency (CCE) of 92% for 5.84 MeV alpha particles (^241^Am source). Additionally, Mulligan et al. [50] have fabricated GaN SBDs (as shown in Figure 5a) for measuring the response for alpha particles (^241^Am source). The 450 µm thick n-type doped GaN wafer with the Ni Schottky contact was prepared. They have obtained excellent results and a charge collection efficiency of 100%.

**Figure 6:**
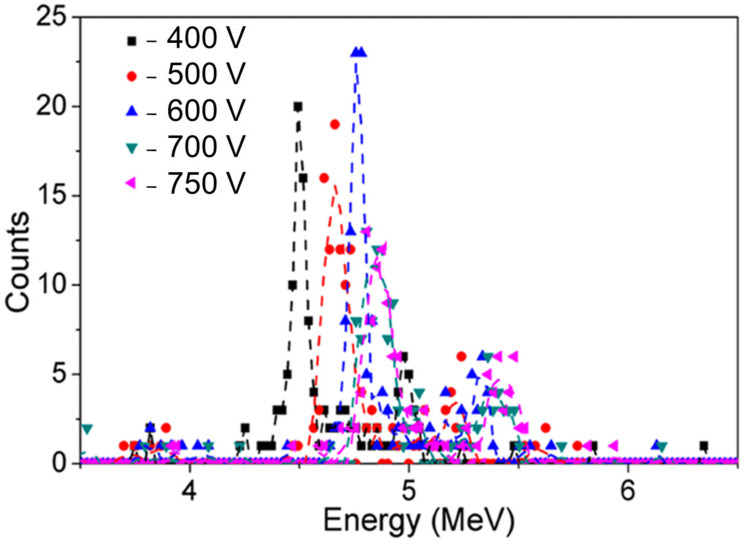
Radiation response of GaN SBD to alpha particles (^241^Am source) at different voltages in vacuum. Data taken from Ref. [51].

Sandupatla et al. [51] reported on GaN SBDs using Ni/Au Schottky contacts with two different GaN thicknesses (15 and 30 µm). Figure 6 shows the radiation response to alpha particles obtained by GaN SBD (30 µm) at different voltages (from −400 up to −750 V) in a vacuum. An excellent CCE of 100% for 5.48 MeV alpha particles was achieved at −750 V [51].

While all the above-mentioned results are obtained at RT, Zhu et al. [52] performed the temperature-dependent (290–450 K) radiation response measurements to alpha particles using the GaN pin device. They observed that the peak maximum is shifted on the x-scale (i.e., energy-scale) as the temperature increases, which is almost identical to what Bernat et al. [33] observed with 4H-SiC SBDs.

The described experiments are summarized in Table 3. For the GaN pin device, thicknesses for all GaN layers are provided.

Another similarity between 4H-SiC and GaN devices is their ability to detect neutrons. Detection could be direct and indirect by using converters made of ^6^Li,^10^B, and ^157^Gd [53]. In the case when neutron converters are used, the main principle is identical to what has been previously described for 4H-SiC SBD (Section 2.1). The converter is placed just above (~4 mm) the Schottky contact (Figure 2b).

**Figure 7:**
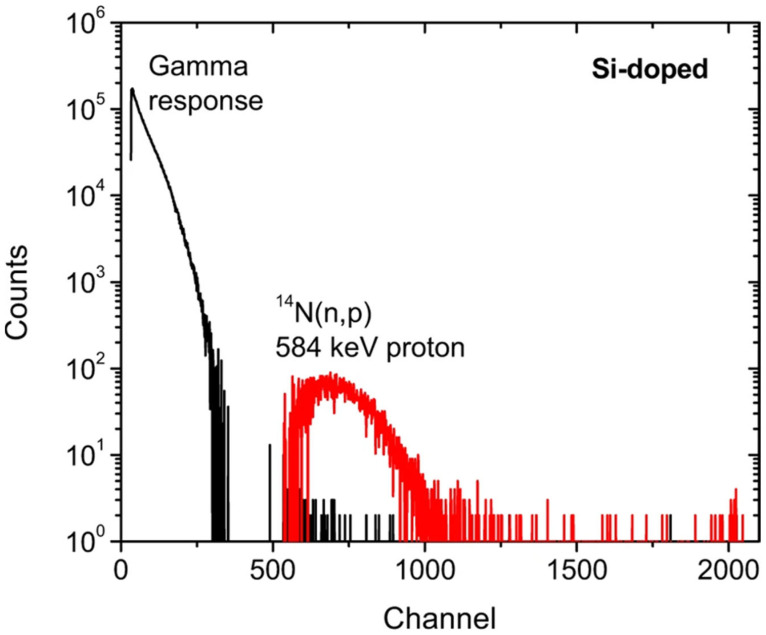
Radiation response to gamma and thermal neutrons measured by Si-doped GaN scintillator without thermal neutron converter. Data taken from Ref. [53].

An interesting prospect that GaN SBD could be used for direct neutron detection through the reaction ^14^N(n,p)^14^C, which could make the neutron converters redundant, has recently been presented by Zhou et al. [53]. Since ^14^N makes up to 50% of the GaN crystal structure, the likelihood of the reaction cannot be neglected. The possibility of using GaN devices without converters would significantly simplify and reduce the costs of the application. Figure 7 shows the gamma and thermal neutron response of Si-doped GaN scintillators exposed to the reactor gamma rays and thermal neutrons. The neutron-induced peak (red spectrum) has been attributed to ionization from 584 keV protons produced by the ^14^N(n,p)^14^C reaction.

The number of published research papers related to the GaN X-ray detectors is noticeably lower compared to the GaN alpha particles or neutron detectors. Here, we mention results obtained by Duboz et al. [54]. They used MSM devices with a 480 µm thick GaN layer and Pt/Au electrical contacts to test radiation response to X-rays. The absorption coefficient in GaN MSM devices was measured as a function of the photon energy in the X-ray range from 6 to 40 keV. They have concluded that GaN could be used as an X-ray detector, but only for energies below 20 keV.

## 4. β-Ga_2_O_3_

**Figure 8:**
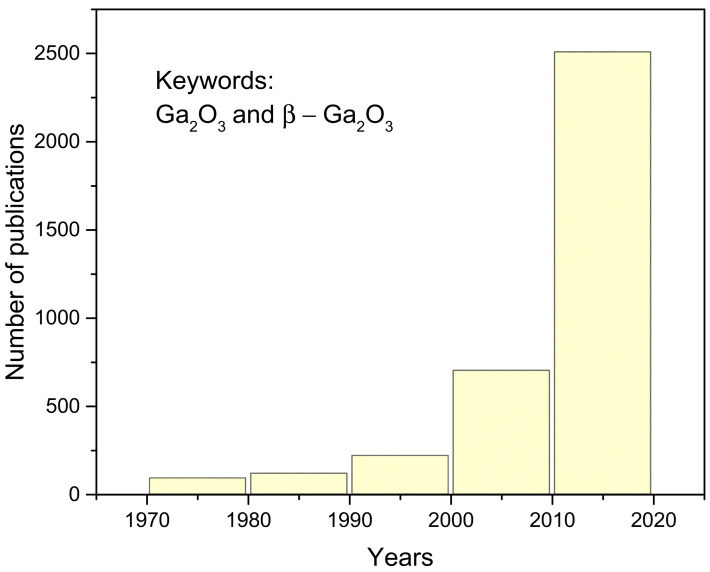
Number of publications on Ga_2_O_3_ from 1970 to 2020. Data are extracted from the Web of Science Core Collection with keywords “Ga_2_O_3_” and “β-Ga_2_O_3_”.

Research on Ga_2_O_3_ shares a similar history as on GaN. Upon the discovery of gallium in 1875, significant improvements were achieved in the 1960s. The band gap of bulk single crystals of Ga_2_O_3_ was estimated as 4.7 eV. In the following decades, the major challenge and limiting factor was the quality of Ga_2_O_3_ crystals [55]. Due to the low crystal quality, the Ga_2_O_3_-based applications were not developed at the same pace as SiC and GaN-based devices. However, Ga_2_O_3_ has attracted significant and fast-growing attention in the past decade, as nicely illustrated by the number of published research papers presented in Figure 8. Among them are recently published review papers on β-Ga_2_O_3_ material properties and crystal growth [55,56].

As previously mentioned, due to the stability and high breakdown electric field, β-Ga_2_O_3_ is the preferred among Ga_2_O_3_ phases and is used for different applications. The number of applications has been continuously growing over the past ten years. β-Ga_2_O_3_ has shown potential for power electronics, solar-blind ultraviolet (UV) photodetectors, and gas sensors [55]. Moreover, like all WBG semiconductors, β-Ga_2_O_3_ has significant potential for applications in harsh environments (high radiation, high temperature, high voltage). Today, the high-power market is dominated by SiC devices, but β-Ga_2_O_3_ is the most promising candidate to take over the ultra-high-power market [57].

Nevertheless, β-Ga_2_O_3_ devices for radiation detection have not yet reached the same level of maturity as 4H-SiC and GaN devices. However, early and very encouraging results on radiation response have been obtained for β-Ga_2_O_3_ X-ray detection. Developing a highly efficient, sensitive, and reliable X-ray detector for medical imaging and homeland security is still a very challenging task. An excellent overview of the recent advancements in β-Ga_2_O_3_-based X-ray detectors and scintillators has recently been published by Prasad et al. [58]. A comparison of the X-ray detector’s crucial parameters such as X-ray-generated photocurrent, response time, response, sensitivity, and signal-to-noise ratio is presented in detail.

**Figure 9:**
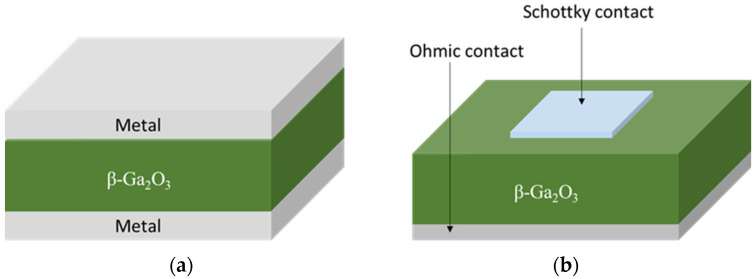
β-Ga_2_O_3_ devices for radiation application: (**a**) MSM and (**b**) SBD.

Figure 9 shows the preferred β-Ga_2_O_3_ devices used for radiation detection: (a) MSM and (b) SBD. Different metals have been used for Schottky contacts like tungsten (W), copper (Cu), nickel (Ni), iridium (Ir), platinum (Pt), and gold (Au) [4]. Pt and Ni are the most used among them.

### Radiation Response to X-rays

Compared to 4H-SiC and GaN, the number of research studies on the radiation response of β-Ga_2_O_3_ devices to alpha particles and/or neutrons is rather limited. However, it is rather impressive to follow the advancements of β-Ga_2_O_3_ devices used for X-ray detection. Here, we will give an overview of the recent results. One of the most used devices is the Fe-doped β-Ga_2_O_3_ MSM. Hany et al. [59] have obtained promising results (response time 0.3 s) and proposed that doping β-Ga_2_O_3_ with Fe could significantly improve the detector performance. They used a 0.5 mm thick Fe-doped β-Ga_2_O_3_ layer with Ti/Au electrical contacts deposited on both sides. Additional improvements were made by Chen et al. [60]. They also fabricated X-ray detectors on Fe-doped β-Ga_2_O_3_ MSM. Again, Ti/Au metal electrodes were deposited on both sides of the Fe-doped Ga_2_O_3_ samples. The detector showed great potential for X-ray detection with a slightly shorter response time (0.2 s) and a high sensitivity.

**Figure 10:**
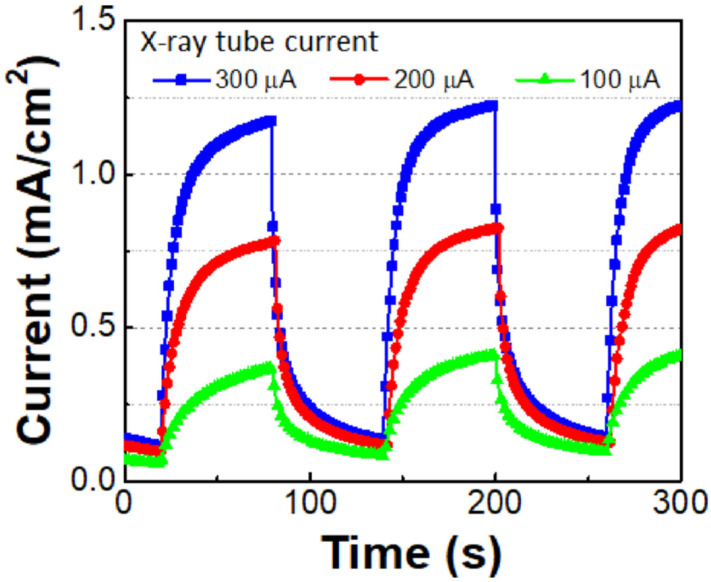
Radiation response of the β-Ga_2_O_3_ SBD to X-ray with different incident flux (controlled by the X-ray tube current). Reprinted from [61] with the permission of AIP Publishing.

Lu et al. [61] investigated the 1mm thick β-Ga_2_O_3_ SBDs with Pt/Au as Schottky contacts for X-ray detection. The X-ray source with a peak photon energy at around 24 keV was used for measuring radiation response. Figure 10 shows the radiation response to different incident fluxes (different flux was achieved by adjusting the X-ray tube current, as shown in Figure 10). This result has again indicated the great potential of β-Ga_2_O_3_ devices for X-ray detection.

Once the fabrication of high-quality β-Ga_2_O_3_ wafers has overcome existing difficulties (such as the presence of electrically active defects), β-Ga_2_O_3_ will exhibit its full potential.

At the end, we will briefly mention a different but still promising approach. Instead of standard devices such as SBD or MSM, Zhang et al. [62] have used Sn-doped β-Ga_2_O_3_ microwires (MWs) to fabricate the X-ray detectors. The Sn-doped Ga_2_O_3_ MWs were synthesized using chemical vapor deposition in a tube furnace and then transferred onto a sapphire substrate. Ag was used for electrical contacts. The fabricated detector exhibits stability over extended temperature ranges, from room temperature to 623 K, which is one of the highest reported operating temperatures for β-Ga_2_O_3_ X-ray detectors.

This direction of research indicates the potential future trends. It is reasonable to expect that more devices based on WBG semiconductor nanostructure (0D, 1D, 2D, and 3D) will gain more attention and enable new advancements [63,64,65].

## 5. Conclusions

The main aim of this paper is to present recent advancements and challenges in the application of WBG semiconductors for radiation detection.

Despite the evident advancements, radiation detection applications of the WBG semiconductors did not reach the same level of development as in the case of power electronics, where 4H-SiC and GaN are the main driving forces. Moreover, the fabrication of high-quality β-Ga_2_O_3_ wafers has yet to overcome several difficulties (for example, defect-free material). Once the crystal quality has improved, the performance of β-Ga_2_O_3_ devices will follow, as was previously the case with 4H-SiC.

It is evident that the most mature technology is related to 4H-SiC and GaN devices, where exceptional results for the detection of alpha particles and neutrons, thermal neutrons in particular, are achieved. Detection of thermal and fast neutrons without additional neutron converters would significantly increase the applications of 4H-SiC and GaN devices. Different 4H-SiC and GaN devices are used, but the most relevant results are obtained with a remarkably simple device such as SBD. However, additional modifications to SBDs, such as the thickness of the epitaxial layer and the choice of metal for the Schottky contact, could allow advances in fast neutron and X-ray detection.

Since 4H-SiC and GaN devices have not yet reached the desired efficiency and energy resolution for the detection of X-rays, that leaves enough space or at least a possible niche for β-Ga_2_O_3_ devices to prosper. The available results on β-Ga_2_O_3_ devices (Fe-doped β-Ga_2_O_3_, in particular) for X-ray detection strongly support this.

The future perspectives for WBG semiconductors are bright. For radiation detection applications, 4H-SiC, GaN, and β-Ga_2_O_3_ form a set of materials that complement each other efficiently and enable the development of detectors that will cover a wide range of radiation (alpha particles, thermal and fast neutrons, X-rays).

## Figures and Tables

**Table 2:** The list of the relevant results and associated 4H-SiC SBDs parameters used for measuring radiation response to alpha particles.

Schottky Contact	Schottky Contact Area (mm^2^)	4H-SiC Epitaxial Layer Thickness (µm)	Energy Resolution FWHM (%)	References
Ni	3.1–7.1	250	0.50	[17]
Ni/Au	0.65	105	0.25	[31]
Ni	3.1–7.1	25, 50	0.29	[22]
Ni	25	25	2.50	[33]

**Table 3:** The list of the GaN devices used for alpha particle (^241^Am source) detection.

Device Structure	Schottky or Electrical Contact	GaN Layer Thickness (µm)	Charge Collection Efficiency (%)	References
Double SBD	Au	2	92	[49]
SBD	Ni	450	100	[50]
SBD	Ni/Au	15, 30	100	[51]
pin	Ti/Al/Ni/Au	2 µm(n)–5 µm(i)–300 nm(p) *	--	[52]

* nGaN–iGaN–pGaN.

## Data Availability

Data are contained within the article.
